# Thermal measurements support a role of the ABA/LANCL1−2 hormone/receptors system in thermogenesis

**DOI:** 10.1098/rsob.240107

**Published:** 2024-12-11

**Authors:** Giovanni Zocchi, Flavio Fontanelli, Sonia Spinelli, Laura Sturla, Mario Passalacqua, José Cristobal González Urra, Simona Delsante, Elena Zocchi

**Affiliations:** ^1^ Department of Physics and Astronomy, University of California, Los Angeles, CA, USA; ^2^ Department of Physics, University of Genoa and National Institute of Nuclear Physics (INFN), Section of Genoa, Italy; ^3^ Laboratory of Molecular Nephrology, IRCCS Istituto Giannina Gaslini, Genoa, Italy; ^4^ Department of Experimental Medicine (DIMES), University of Genoa, Genoa, Italy; ^5^ Instituto de Quimica, Pontificia Universidad Catolica de Valparaiso, Chile; ^6^ Department of Chemistry and Industrial Chemistry (DCCI), University of Genoa, Genoa, Italy

**Keywords:** heat production by cells, ABA, Lancl Proteins, thermistor probes, DSC, H9c2 cardiomyocytes

## Abstract

Abscisic acid (ABA) is a conserved ‘stress hormone’ in unicellular organisms, plants and animals. In mammals, ABA and its receptors LANCL1 and LANCL2 stimulate insulin-independent cell glucose uptake and oxidative metabolism: overexpression of LANCL1/2 increases, and their silencing conversely reduces, mitochondrial number, respiration and proton gradient dissipation in muscle cells and in brown adipocytes. We hypothesized that the ABA/LANCL hormone/receptors system could be involved in thermogenesis. Heat production by LANCL1/2-overexpressing versus double-silenced cells was compared in rat H9c2 cardiomyocytes with two different methods: differential temperature measurements using sensitive thermistor probes and differential isothermal calorimetry. Overexpressing cells generate an approximately double amount of thermal power compared with double-silenced cells, and addition of ABA further doubles heat production in overexpressing cells. With the temperature probes, we find a timescale of approximately 4 min for thermogenesis to 'turn on’ after nutrient addition. We provide direct measurements of increased heat production triggered by the ABA/LANCL hormone receptors system. Combined with previous work on oxphos decoupling, these results support the role of the ABA/LANCL hormone receptors system as a hitherto unknown regulator of cell thermogenesis.

## 1. Introduction

The capacity to adapt to environmental challenges is as fundamental a feature of the living as is the ability to reproduce. Abscisic acid (ABA) is a terpenoid signal molecule present in unicellular organisms to higher plants and mammals, with a conserved, cross-kingdom role as a ‘stress signal’, allowing organisms to respond to environmental stimuli as diverse as water and nutrient availability, blood glucose levels, UV irradiation [1].

ABA is produced in response to water and nutrient availability by microalgae, as well as by higher plants [2], to UV light by plant stomatal cells, as well as by human granulocytes and keratinocytes [3], to hypoxia by higher plants, as well as by mammalian cardiomyocytes [4,5]. Such diversity of cell types and functional responses is obviously mediated by cell-specific signaling pathways and molecular effectors, however; ABA emerges as a hormonal signal orchestrating the response to environmental stress of organisms as distant in evolution as marine sponges [6] and mammalian cardiomyocytes [5]. It should be noted that the environmental stimuli eliciting ABA-mediated responses are very ‘basic’: soil humidity, nutrient and oxygen availability affecting plant growth, water temperature regulating marine sponge filtration, UV light inducing plant antioxidant defences and human keratinocyte inflammatory response.

ABA perception occurs through different receptors in plants [7] and in animals. In mammals, two ABA receptors are known so far, LANCL1 and LANCL2, belonging to the lanthionine synthetase C-like (LanCL) gene family, most likely the result of an ancestral gene duplication, which also contains LANCL3, transcribed at very low levels and probably a pseudogene. LANCL proteins show a significant sequence homology with bacterial lanthionine synthetase, which produces cysteine-derived natural antibiotics (lantibiotics). Although mammalian LANCL proteins do not synthesize lantibiotics [8], they evidently play an essential role in animal physiology since triple LANCL knock-out (KO) mice die prematurely [9]. LANCL1 and LANCL2 share several structural and functional features which make them a redundant system for ABA sensing and signalling: (i) both are attached to the inner side of the plasma membrane, through which ABA is transported by non-specific anion transporters [10]; (ii) they are similarly and ubiquitously expressed in mammalian tissues and organs (https://www.proteinatlas.org/); (iii) both bind ABA, albeit with a somewhat different affinity, with a Kd in the submicromolar and in the low micromolar range, respectively [11,12] and (iv) they both activate the AMPK/PGC−1α/ERRα/Sirt1 axis, a signalling pathway that controls mitochondrial mass and function. Interestingly, silencing of LANCL2 in cells or its genetic ablation in mice induces the spontaneous overexpression of LANCL1, while silencing of LANCL1 results in overexpression of LANCL2 in adipose tissue and muscle cells [12,13]. Receptor redundancy and a reciprocal ‘compensatory’ transcriptional control again point to the physiological relevance of the ABA-LANCL1/2 hormone-receptor system in mammals.

Indeed, the ABA/LANCL system has been shown to stimulate insulin-independent glucose uptake and its oxidative metabolism in adipocytes and muscle cells, which, combined, represent approximately 50% of mammalian body weight. As a consequence of this ABA/LANCL-mediated increase of glucose uptake and metabolism by muscle and adipose tissue, blood glucose levels are reduced in ABA-treated rodents and humans as compared with untreated controls [14,15]. The AMPK/PGC−1α/ERRα axis activated by the ABA-LANCL1/2 hormone-receptors system not only controls glycaemia, by stimulating adipose and muscle cells to drain glucose from the blood, but also stimulates mitochondrial respiration in muscle and adipose cells [12,13]. Mitochondria are the power plant of cells, where enzymatic oxidation of metabolic substrates produces reduced nicotinamide adenine dinucleotide (NAD) and flavin adeninde dinucleotide (FAD) coenzymes, which in turn power the respiratory chain, generating the mitochondrial proton gradient (ΔΨ) between the inner mitochondrial membrane and the mitosol that eventually allows ADP phosphorylation to ATP by ATP synthase. The partial dissipation of the ΔΨ by specific proton-channels allows thermogenesis, arguably as essential a feature of the animal kingdom as respiration itself. These proton channels are referred to as uncoupling proteins, because they partly uncouple proton flux through the inner mitochondrial membrane from ATP synthesis. They are under the transcriptional control of specific hormones that regulate thermogenesis, i.e. T3 and catecholamines, acting via specific receptors (thyroid hormone and β-adrenergic receptors, respectively). Recently, overexpression of LANCL1 and/or of LANCL2 was shown to increase, while their combined silencing conversely reduced, mitochondrial number, respiration and uncoupling both in human brown adipocytes [13] and in rat cardiomyocytes [5]. Notably, overexpression of LANCL1/2 *per se* (without addition of ABA) induced these significant effects on mitochondrial function, by activating a transcriptional response leading to activation of the AMPK/PGC−1α/Sirt1 axis, which in turn controls mitochondrial biogenesis and function [16]. The significantly higher mitochondrial mass, O_2_ consumption and oxidative phosphorylation (oxphos) uncoupling observed in LANCL1/2 overexpressing rat cardiomyocytes should result in higher heat production of the overexpressing compared with the double-silenced cells under the same conditions; however, a direct temperature or heat production measurement on cells has never been performed until now.

The aim of this study was to directly measure heat production by LANCL1/2-overexpressing versus double-silenced H9c2 rat cardiomyocytes with super-sensitive temperature probes on relatively small cell samples. These measures were compared with those obtained with a differential scanning calorimeter (DSC) on the same cell types. Results obtained indicate an almost double heat production by LANCL1/2-overexpressing versus double-silenced cells, and a further 100% increase of heat production in ABA-treated versus -untreated LANCL1/2-overexpressing cells. These results provide the first direct demonstration that the ABA-LANCL1/2 hormone-receptors system triggers heat production in mammalian cells. Taken together with previous studies on oxphos decoupling, the present study indicates a role for the ABA-LANCL system in the control of cell thermogenesis.

## 2. Material and methods

### 2.1. Cell transduction

H9c2 rat cardiomyoblasts, obtained from ATCC (LGC Standards s.r.l. Milan, Italy), were cultured in Dulbecco’s modified Eagle’s medium (DMEM) containing 25 mM glucose (DMEM-high glucose) (Sigma-Aldrich, Milan, Italy) supplemented with 10% fetal bovine serum (FBS) (Sigma-Aldrich, Milan, Italy), penicillin (62.5 μg ml^−1^) and streptomycin (100 μg ml^−1^) (Sigma-Aldrich, Milan, Italy) (complete medium) in a humidified atmosphere containing 5% CO_2_ at 37°C.

LANCL1 and LANCL2 silencing (sh), LANCL1 and LANCL2 overexpression (ov) and cell transductions were performed as described in [12].

The protein expression levels of hLANCL1 and hLANCL2 in over-expressing cells were approximately 10 and 40 times higher than in control cells transfected with the empty vector, while protein levels were both reduced by approximately 90% in double-silenced cells, as detected by Western blot and qPCR (see figure and Methods in the electronic supplementary materials), in line with previously published values [5].

### 2.2. Cell culture

Twenty-four hours before each experiment, cells were seeded at a density of 1×10^6^ in 75 cm^2^ flasks in DMEM containing 5 mM glucose (DMEM low glucose), without FBS. ‘Starved’ cells were harvested by brief trypsin treatment, which was less traumatic to cells than scraping, washed twice in PBS containing Ca^2+^/Mg^2+^and resuspended in the same buffer at 12.5×10^6^cells ml^−1^ (for DSC experiments) or at 2.5×10^6 ^cells ml^−1^ for measures with the temperature probes. Cells were kept at room temperature in the immediate vicinity of the instrument to be used for temperature recording (DSC or temperature probes set-up) for thermalization until use.

The final concentrations of the nutrients used to start oxidative metabolism in the starved cells were the same in all experiments: 2 mM glutamine, 1 mM pyruvate and 17.5 mM glucose in PBS with Ca^2+^/Mg^2+^. These nutrients provide the necessary substrates to the Krebs cycle (glutamine and pyruvate) and to glycolysis (glucose) to allow the cells’ mitochondrial oxidative metabolism to restart after starvation. The indicated concentrations of Gln, pyruvate and glucose are the standard concentrations present in culture media, such as DMEM, and are approximately three, eight and three times higher, respectively, than those present in normal human serum in the fasting state. To inhibit oxidative phosphorylation, cells were pre-incubated for 2 h with 0.03 mg ml^−1^ digitonin, 0.5 µM rotenone and 0.5 µM antimycin A prior to experimental measures. To inhibit glycolysis and mitochondrial respiration, 3 mM KCN and 10 mM 2’-deoxy-glucose were added together with the nutrients immediately before measurements.

### 2.3. Temperature probes

NTC type BR series, glass encapsulated bead thermistors cat. no. BR55KA622K, produced by Amphenol were obtained from Mouser Electronics, Inc., Milano, Italy. The glass bead has a nominal diameter × length of 1.4×3 mm and a thermal time constant of 0.2 s in water. The temperature coefficient (% resistance change / °C) is α=-4.0 at 25°C, and we used probes with resistance R≈6.2 kΩ at room temperature. Some measurements were also obtained with epoxy encapsulated NTC type thermistors produced by Vishay (cat. no. NTCLE305E4502SB) obtained from Newark Electronics (cat. no. 86R9240). These have similar characteristics: α=-3.7 at 25°C, R≈5 kΩ at room temperature. After carefully soldering the leads to insulated copper wire, the former were mounted on a 1 ml type disposable pipette tip, cut to size to allow the wires to run inside the pipette tip, with the glass bulb encapsulating the thermistor protruding at the end. The thermistor leads were glued to the pipette tip, for mechanical stability and electrical insulation, using 5 min epoxy. This insulation proved unreliable in water after about 20 min, which restricted our measurements to timescales below 10 min, the probe assemblies being air dried in between measurements. The epoxy-encapsulated probes were suspended directly by their (factory insulated) leads from the screw cap of a 1.5 ml vial. For the measurements, the two probes (corresponding to sample and reference) were wired as two arms of a Wheatstone bridge configuration, thus automatically taking the difference signal between the probes. The other two arms of the bridge were formed by 6.7 kΩ resistances and a 1 kΩ, 10 turns potentiometer which allowed the bridge to be balanced so the difference signal could be amplified. We used lock-in detection, the bridge being powered by the sinusoidal reference signal from a lock-in amplifier (Stanford Research Systems, model SR530).

The signal was acquired by a PC running National Instruments Labview software via a PCI6321 data acquisition board.). Typical settings for the lock-in amplifier were: reference signal $V_{in}=140$ mV frequency, 500 µV sensitivity, time constant. The potentiometer on the bridge then allowed the signal to be zeroed within a few microvolts before starting the measurements. From the bridge configuration and the temperature coefficient of the probes, with $V_{in}=100$ mV, a 10 µV signal corresponded to a temperature difference of 0.01°C.

### 2.4. Experimental procedure with the temperature probes

All parts of the set-up were pre-thermalized at room temperature for at least 30 min before starting the experiments. In a typical experiment, 1.0 ml of a cell suspension containing $2.5\times 10^{6}$ cells in PBS with Ca^2+^/Mg^2+^ was added to each one of two Eppendorf tubes, held in a holder, in air. With the probes in the air, the signal was zeroed by means of the potentiometer. Then, a temperature probe assembly was inserted in each Eppendorf, the micropipette tip being held in position at the centre of the suspension by a removable Styrofoam lid. The recording was started immediately after insertion of the probes in the cell suspension; after thermalization of the probes, the probes were removed, a volume of 10 µl of PBS, containing or not the nutrient mixture to allow cell oxidative metabolism, was added to the Eppendorf tubes, the cell suspension was mixed once by gentle pipetting, the probes were inserted again and fixed in the central position of the suspension and recordings were started again.

### 2.5. Differential scanning calorimetry

Measurements were performed using a heat flux differential scanning calorimetry (DSC 111 by Setaram) designed as a Calvet-type calorimeter. The sample and reference sensors are inserted in a calorimetric block and are composed of 120 thermocouples mounted in a cylinder surrounding the measurement zone and providing a highly sensitivity sensor.

Briefly, 200 µl of the cell suspensions to be compared were added to each one of two ‘home-made’ Ta crucibles and thermalized at ambient temperature for at least 30 min. After addition of the nutrient mixture (10 µl), or of an equal volume of PBS with Ca^2+^/Mg^2+^ (controls) the crucibles were capped, placed inside the DSC chamber and the temperature ramp program was started. A typical temperature recording is shown in figure 5. Recordings were acquired for 2450 s.

## 3. Results

In-depth transcriptional and metabolic investigations performed in previous studies allowed us to conclude that LANCL1/2 double-silenced H9c2 cells showed a significantly reduced, and over-expressing cell, conversely a significantly improved, transcriptional and functional phenotype compared with the respective control (cells transfected with the scrambled sequences used for LANCL1/2 silencing or infected with the empty vector used for LANCL1/2 over-expression). In particular, we studied the signalling axis activated by the ABA-LANCL system (AMPK, PGC−1α, ERRα and Sirt1), metabolic pathways (glucose transporters, glycolytic and oxidative metabolism enzymes, NAD^+^ and NO synthesis), structural and cell cycle regulatory proteins (cytoskeletal proteins, cyclins and cyclin-dependent kinases, cell doubling time) and mitochondrial function (proton gradient, respiration). Moreover, double-silenced cells did not respond to ABA with any significant activation of these functions, as conversely occurred in overexpressing cells [5,17]. For these reasons, in the present study, we focused on the direct comparison between double-silenced and overexpressing cells, as the experimental systems used for heat measurement allowed two samples at a time to be compared.

### 3.1. Thermistor-based measurements

#### 3.1.1. Temperature probe experiments

Experiments were performed in the following way. Two empty Eppendorf tubes and two temperature probe assemblies were prepared and thermalized in air next to each other. The Eppendorf tubes were in a plastic holder, about 5 cm apart, in air. The probes were held by a polystyrene ‘lid’, which allowed them to be plunged simultaneously into the Eppendorf tubes. At this point, the bridge was (approximately) balanced by means of the 10 turns potentiometer, and not touched again. Two further cell suspensions, to be used in the following experiment, were also prepared beforehand in Eppendorf tubes and left to thermalize in the vicinity of the experimental set-up. Recording of the temperature signal was started and after a few seconds, when the signals were stable, 10 µl of the nutrient solution in PBS+Ca^2+^/Mg^2+^, or of PBS+Ca^2+^/Mg^2+^ without nutrients (control), was pipetted into the cell suspensions and the suspensions mixed once by gentle pipetting. Immediately afterwards the probes were plunged into the tubes, and a plastic box was placed over the experiment to reduce disturbances due to air currents. The ‘dead time’ between mixing in the last component and the end of trace disturbances allowing the start of useful measurements was about 20 s (i.e. relatively short compared to the characteristic timescales of the experiment, as we shall see).

**Figure 1.**
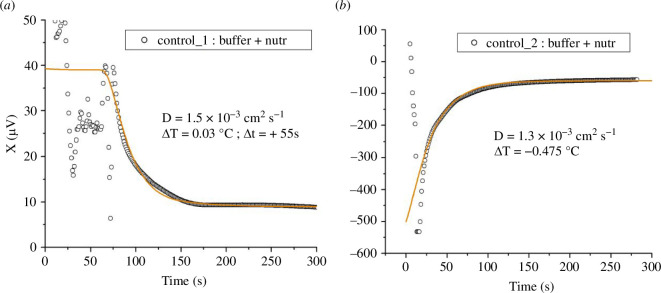
Thermal relaxation signals measured in control Eppendorfs, containing buffer with nutrients alone. (*a*) Thermal relaxation signal reflecting an initial small temperature difference ΔT=0.03°C between the sample and reference vials. Both vials contain the same 1ml volume mixture of buffer and nutrients. The output of the lock-in amplifier X is shown in the course of time; 10 μV correspond to 10mK in temperature. The data are well represented by the model described in the text (solid line), with zero heating power (w=0) and an effective diffusion constant ($D=1.5\times 10^{-3}\;\;cm^{2}\;s^{-1}$) equal to that of water. (*b*) Thermal relaxation signal corresponding to a negative initial temperature difference $\Delta T=-\;0.475{\;}^{\circ }C$ between the sample and reference vials. The solid line shows the model with the same parameter values as in (*a*), except for ∆T and a slightly smaller diffusion constant $D=1.3\times 10^{-3}\;cm^{2}\;s^{-1}$ (see text for explanation).

When the probes were plunged into the Eppendorf tubes there were transient large fluctuations in the signal, followed by an exponential relaxation to a steady state. The reason is an initial, unavoidable small temperature difference (of the order of a few hundredths of degrees centigrade) between the two samples, which was introduced during mixing and transferring. Figure 1 shows two examples of this relaxation process.

In both cases, the two Eppendorfs (sample and reference) contained identical solutions (buffer + nutrients). We plot the output X of the lock-in amplifier in the course of time, where 10 µV corresponds to a temperature difference between the vials of approximately 0.01°C. Because the bridge is not perfectly balanced, only differences in the signal X are significant, not the absolute value. For figure 1*a*, the initial temperature difference between vial 1 (reference) and vial 2 (sample) was positive and approximately equal to 0.03°C, i.e. $\Delta T=T_{2}-T_{1}\approx +$0.03°C. The corresponding total excursion of the signal over the relaxation process is about 30 µV. The heat diffusion time characteristic of the system can be obtained by fitting an exponential relaxation to these data, and is found to be about 30 s (fit not shown). The solid line in the plot is the result of solving the diffusion equation for an idealized model of the experimental system, which we discuss later.

Figure 1*b* shows a case where the initial temperature difference between the vials was negative and much larger ΔT≈−0.475°C. This large mismatch originated from the different initial temperatures of the probe assemblies: these were wet from a previous measurement and partially blow-dried in air, resulting in relatively large temperature variations across the assembly due to uneven water evaporation. Also, the initial steep variation of the signal reflects the lock-in recovering from overload below − 500 µV. However, the same model (solid line) accounts for the relaxation to the steady state, and the relaxation time is essentially the same as in figure 1*a*, as expected. The slightly different diffusion constant ($1.3\times 10^{-3}$ cm^2^  s^−1^) used in figure 1*b* reflects the increased role of heat transfer between the probe assemblies and the solutions.

**Figure 2.**
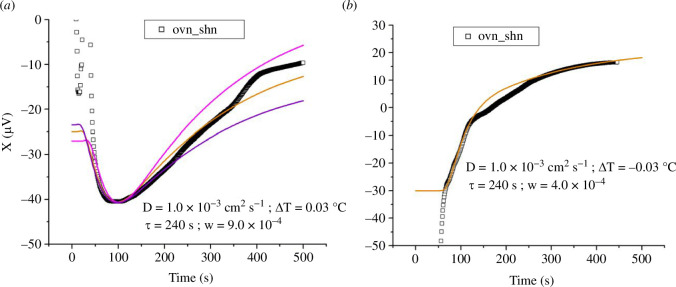
Time course of the temperature variation between LANCL1/2-overexpressing and double silenced H9c2 after addition of nutrients. (*a*) Time course of the temperature variation measured for a sample of LANCL1/2 overexpressing cells with nutrients (ovn); the reference vial contained double-silenced cells with nutrients (shn). A 10 μV signal corresponds to a temperature variation of 0.01°C. With a positive initial temperature difference between sample and reference vials, the initial thermal relaxation corresponds to a decrease in the signal, followed by an increase due to additional heat produced by the cells in the sample vial. The ochra solid line represents the model (see text) with parameter values $w=9.0\times 10^{-4}\;\;cal\;K^{-1}$, $D=1.0\times 10^{-3}cm^{2}\;s^{-1}$, ΔT=0.03°C and τ=240s. The magenta and purple lines are drawn in for comparison and represent the same model with $w=11.0\times 10^{-4}cal\;K^{-1}$ and $w=7.5\times 10^{-4}cal\;K^{-1}$, respectively. The ochra line corresponds to a steady state heating power produced by the cells of $P=9.0\times 10^{-4}cal\;s^{-1}=3.8\;mW$ according to the model; τ is the characteristic timescale for the cells to ‘turn on’ after addition of the nutrients. (*b*) Measurement of the temperature variation for a sample similar to the sample of figure 2*a* (LANCL1/2 overexpressing cells vs double-silenced cells). In this case the initial temperature difference between sample and reference vials was negative, leading to a monotonic increase of the signal in the course of time. The first approx. 50 s of this increase (corresponding to −30<X<0 [μV]) is due to thermal relaxation of the initial temperature difference; the subsequent signal increase (corresponding to 0<X<15 [μV]) is due to additional heat produced by the cells in the sample vial. The solid line shows the model with the same parameter values as in (*a*) except for $w=4.0\times 10^{-4}cal\;K^{-1}$ and ΔT=−0.03°C.

The experiments with cells were performed with the cell suspension to be tested in the test vial (vial 2), and the control cell suspension in the reference vial (vial 1), so that ΔT>0 means that additional heat is being released by the cells to be tested compared to the control. Figure 2 shows two examples of cells overexpressing LANCL1/2 (ov), compared to double-silenced cells (sh), both vials containing nutrients.

In the following, we label this configuration as ‘ovn_shn’. In figure 2*a* the initial temperature difference was positive (ΔT≈0.03°C), similar to figure 1*a*. However, the initial relaxation, which plateaus at X≈−40 μV (t≈100s) is now followed by a monotonic increase, which signifies additional heat released in vial 2. If the cells were releasing heat at constant power, we would expect the signal to plateau at long times, reflecting the steady state temperature difference resulting from a steady state heat source in vial 2. The magnitude of the power emitted determines the signal difference ∆X between the minimum (X≈−40 μV) and the plateau at long times in the measurement (X≈−10 μV, determined from the ochra line in the plot). In the case of figure 2*a*, ΔX≈30 μV corresponding to a steady state temperature difference $∆T_{f}\approx 0.03C$ and ultimately a power per unit volume emitted $P\approx 9.0\times 10^{-4}\;cal\;{\left(cm^{3}s\right)}^{-1}$, as we see below. The time course of the signal for t>100s, on the other hand, informs on the dynamics of the cells ‘turning on’ after nutrients are added, as we shall see.

Figure 2*b* shows a measurement on a similar sample, where the initial temperature difference was negative, similar to figure 1*b*. In this case the signal is monotonically increasing because both the initial thermal relaxation (over a approx. 30 s timescale, here corresponding to −30<X<0 μV) and the heating by the cells lead to an increase in the signal X(t). We obtained several measurements with LANCL1/2 overexpressing cells versus silenced cells, using $N=2.5\times 10^{6}$ cells in a V=1ml volume; they all show an additional heating power emitted by the overexpressing cells, compared to the silenced cells. Quantitatively, if we use the model of §1.2 to interpret the temperature measurements, we find this additional steady state heating power to be approximately $P\approx 8.0\times 10^{-4}\;cal\;s^{-1}=3.3$ mV. However, the experimental calibration of §1.3 provides a more realistic estimate for the heating power, P≈450 μW for the trace of figure 2*a* and P≈300μW for figure 2*b*.

On the other hand, the model also provides an estimate for the typical timescale τ for the cells to metabolically ‘turn on’. From the time traces of figure 2, we find τ≈4min.

**Figure 3.**
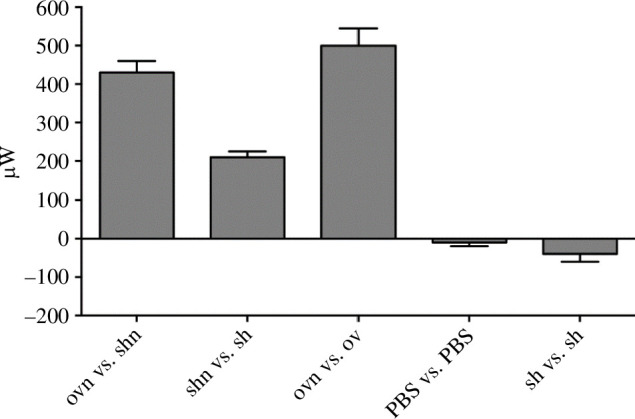
Summary of the experiments with the temperature probes.

For the bar graph shown in figure 3, we used the experimental calibration to obtain the values of the heating power emitted.

The power produced by 2.5 × 10^6^ LANCL1/2-overexpressing (ov) or double-silenced (sh) cells, with (*n*) or without nutrients was comparatively measured as described in par. 1.1. At least one control was always performed alongside each experimental measure. The results shown are the mean ± s.d. of the power emission from at least three different measures for each experiment.

#### 3.1.2. Model

In order to interpret the experimental time traces obtained with the thermistor probes, it is useful to refer to a model of the heat exchange in the experiments. In physical terms, the experimental system consists of a distributed heat source (the cells) in 1 ml of water, in a thermally conductive container (the EP tube) with outer walls at the temperature of the room. Detailed in electronic supplementary material, we consider the following simplified description of the system: a uniform sphere of radius *R*, with thermal diffusion constant *D*, uniformly heated by a (spherical and concentric) distributed heat source of radius $R_{c}<R$ and power per unit volume P(t). The experiments show that it is necessary to attach a time dependence to P, because the cells do not ‘turn on’ instantaneously when nutrients are added at t=0. The outer surface of the sphere is held at temperature $T_{1}$ (the ambient temperature), and we solve the initial value problem for the corresponding diffusion equation, with an initially uniform temperature distribution $T=T_{2}$ for r≤R (r is the radial coordinate, $T\left(r,t\right)$the temperature). In spherical coordinates, and with the substitution $rT\left(r,t\right)\equiv \theta (r,t)$, the diffusion equation for this problem reads (see electronic supplementary material):


$$\frac{\partial \theta \left(r,t\right)}{\partial t}-D\;\frac{\partial^{2}\theta }{\partial r^{2}}=w\;r\;\left[1-H\left(r-R_{c}\right)\right]\;\left(1-e^{-\frac{t}{\tau }}\right)$$

where $w=P/(\rho c_{V})$, ρ is the density, $c_{V}$ the specific heat, H the step function, and we assume that the cells ‘turn on’ exponentially in time with a characteristic timescale τ. We solve this equation numerically using Mathematica, and compare the solutions to the data by ‘manually’ adjusting the parameters (see electronic supplementary material). The effective diffusion constant D is obtained from the thermal relaxation process in the control experiments where the two vials contain identical, non-active solutions. The solid line in figure 1*a* shows the solution of the above equation with w=0, initial temperature difference $∆T=T_{2}-T_{1}=0.03℃$, and $D=1.5\times 10^{-3}\;cm^{2}\;s^{-1}$. Reassuringly, the value obtained for *D* is the diffusion constant of water ($D_{w}=1.5\times 10^{-3}\;cm^{2}\;s^{-1}$ at room temperature). Similarly, the solid line in figure 1*b* is obtained with w=0, ∆T=-0.475℃, and $D=1.3\times 10^{-3}\;cm^{2}\;s^{-1}$, a value within 15% of the value found from figure 1*a*.

The experiments with overexpressing cells can now be analysed with the model with w>0. For the cell suspensions, good agreement between model and experiment is obtained using a diffusion constant $D=1.0\times 10^{-3}\;cm^{2}\;s^{-1}$. In figure 2*a*, the solid ochra line is obtained for $D=1.0\times 10^{-3}\;cm^{2}\;s^{-1}$, ∆T=0.03C, τ=240s and $w=9.0\times 10^{-4}cal\;K^{-1}$. We use $c_{V}=1\;cal\;{\left(g{\;}^{\circ }K\right)}^{-1}$ and $\rho =1\;g\;cm^{-3}$, then w is numerically equal to the power per unit volume P, in $cal\;{\left(cm^{3}s\right)}^{-1}$. The other two lines in figure 2*a* show the effect of varying the parameter w by ~20% : $w=11.0\times 10^{-4}cal\;K^{-1}$ for the upper (magenta) line and $w=7.5\times 10^{-4}cal\;K^{-1}$ for the lower (purple) line. In figure 2*b*, the solid line shows the model with the same parameter values as for figure 2*a*, except the initial temperature difference, which is now negative (∆T=-0.03C) and the heat emitted, which is smaller ($w=4.0\times 10^{-4}cal\;K^{-1}$). The model above correctly describes the time course of the temperature measurements, thus reassuring us that the interpretation of the measurements is qualitatively correct. However, the experimental calibration of the next section shows that this model overestimates the heating power deduced from the temperature traces. The reason is the simplified boundary condition adopted in the model (fixed temperature at the surface of the sphere, corresponding to the outer EP surface). In reality, the heat transfer at the outer surface presumably occurs through a boundary layer in air, and is less than what would occur if the temperature of the EP outer surface was truly fixed.

#### 3.1.3. Experimental calibration

We performed a series of experiments to directly establish the time course of the temperature signal corresponding to a known heating power in the ‘sample’ vial. To this end, a 10 Ω resistance was suspended by its leads near the bottom of one of the experimental vials, and used as a heater. The resistance was powered by a DC power supply through a voltage divider, so that 0.5 V at the power supply corresponded to 250 µW heating power. Otherwise, the set-up was equivalent (but, for logistics reasons, not identical) to the one used in the experiments with the cells. Among the differences was that the vials, in air, were enclosed in an Al box, itself enclosed in a bigger styrofoam box. The purpose was to also determine the long-term stability of the measurements. The calibration measurements were performed with 1 ml of 0.1 M salt solution in both vials, and similar settings for the lock-in amplifier as before (200 mV excitation at 185 Hz, 200 µV sensitivity, time constant 1 s). Time traces were aquired through the analogue output of the lock-in amplifier, digitized at 10 Hz by a NI ADC, controlled by a Labview program.

**Figure 4.**
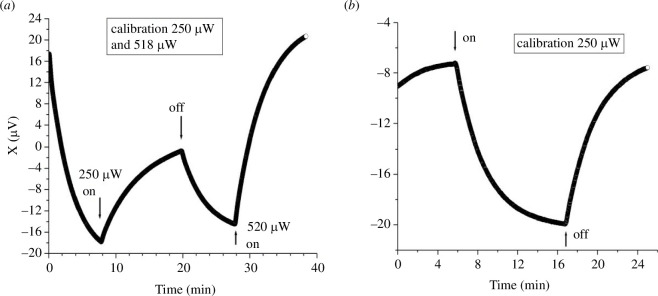
Calibrations. (*a*) Calibration experiment performed with a heating resistance inside the sample vial, simulating the heat produced by the cells. The initial drop in the signal corresponds to thermalization of an initial temperature difference between the vials. At t≈8min the heating resistance is turned on, delivering P=250 µW. At t≈20min the resistance is turned off, and at t≈28min it is turned on again, delivering P=518 µW. (*b*) Calibration experiment showing the temperature signal produced by the heating resistance delivering P=250 µW to the sample vial. The heating was turned on at t≈6min and off at t≈17min. The sample and reference vials were inverted with respect to figure 4*a*.

Figure 4*a* shows an example of experimental calibration. The initial drop in the signal for 0 < *t* < 8 min corresponds to thermalization of an initial temperature difference between the vials. At t≈8min (before thermalization is complete, in this case) the heating is turned on in the ‘sample’ vial, with P=250 µW. It is turned off at t≈20min, whence the temperature relaxes back (20<t<20min). At t≈28min the heating is again turned on, with P=518 µW, giving rise to a larger temperature signal.

Figure 4*b* shows another example of calibration, with the system better thermalized initially, and P=250 µV. The configuration was slightly different here, with the two vials closer to each other and wrapped in Al foil, which produces a somewhat smaller signal for the same heating power. From these experiments we obtain that in our set-up, a signal ΔX=10 µV in the steady state corresponds approximately to a heating power P=150 µV. We used this figure to convert the temperature measures to heating power for the results summarized in figure 3.

### 3.2. Differential isothermal calorimetry

Preliminary experiments performed with a temperature ramp increasing from 22°C to 24°C in 2450 s showed a downward sloping trace with controls (empty crucibles, or crucibles containing PBS), which made it difficult to identify a possible thermal difference between the crucibles once cells replaced the controls. Thus, we performed all measures under isothermal conditions, at a set temperature of 22°C.

**Figure 5.**
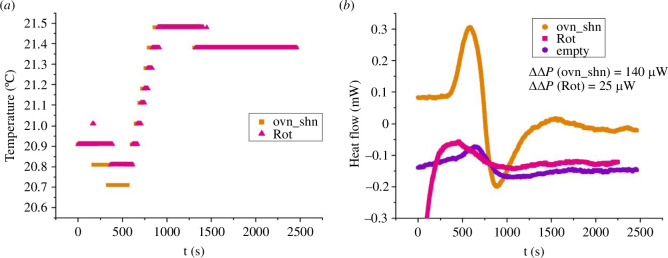
Isothermal DSC measurements for three different experiments: overexpressing cells with nutrients versus silenced cells with nutrients (ovn_shn), overexpressing cells with nutrients and oxphos inhibitors rotenone and antimycin A (Rot) versus silenced cells with nutrients, and empty crucibles (empty). The temperature was set at a nominal 22°C, slightly above the temperature of the room.(*a*) The time course of the temperature shows the action of the temperature controller, the temperature being stabilized for t>800s. (*b*) The time course of the heat flow (in milliwatts; positive for heat flowing out of the crucibles) shows a characteristic ‘up-down’ signature in the first ~800s, corresponding to the action of the temperature control feedback mechanism. The time beyond which the temperature is stable (t=800s) corresponds to the minimum of the traces. The rise beyond this minimum is a measure of the differential heating power in the sample, which we call ΔP. For the ‘empty’ trace, which serves as reference, ΔP[empty]=18 μW, whereas ΔP[ovn_shn]=184 μW and ΔP[Rot]=19 μW. At long times (t>1500s) the heat flow is essentially constant; this steady state value, minus the reference value of the ‘empty’ trace (which represents an offset of the instrument), is also a measure of the heating power in the sample, which we call ΔΔP. From the ochra and purple traces we find ΔΔP[ovn_shn]=140 μW, while from the magenta and purple ΔΔP[Rot2h]=25 μW. This example shows that both the ΔP and the ΔΔP measures agree in finding that the heating power produced by the overexpressing cells is approximately 150 μW larger than the heating power of the silenced cells. By contrast, the heating power of the oxphos inhibited cells is essentially zero within our resolution (19−18=1 μW by the ΔP measure and 25 μW by the ΔΔP measure).

The DSC instrument actively controls the temperature and measures the heat flow (positive for heat flowing out of the crucible), taking the difference between the ‘sample’ and ‘reference’ crucibles. For our isothermal measurements near room temperature, once the samples are locked in, the temperature controller takes about 15 min to stabilize the crucibles at the set temperature. Figure 5 shows representative time traces for three different experiments, which we identify by the following shorthand. Ovn_shn means that the sample crucible contains overexpressing cells with nutrients while the reference crucible contains silenced cells with nutrients. Empty means that both crucibles are empty. Rot means that the sample crucible contains overexpressing cells with nutrients, and rotenone and antimycin A (to inhibit oxidative phosphorylation), while the reference crucible contains silenced cells with nutrients. The action of the temperature controller is visible in the temperature recording of figure 5*a*, where the nominally set temperature was 22°C, while the initial (room) temperature was about 1°C lower. The heat input (positive or negative) due to the temperature controller adjusting the temperature to the set value is reflected in the oscillation visible in the initial part of the time traces (0<t<1000s approximately) of figure 5*b*. The non-zero signal even in the case of the ‘empty’ trace is due to inevitable small differences in the two crucibles’ thermal assemblies. In particular, the average negative heat flow for the ‘empty’ trace represents an offset of the instrument. In this example, the temperature stabilizes at t≈800s (figure 5*a*); this time corresponds to the local minimum in the heat flow traces of figure 5*b*. Thereafter, the ‘empty’ trace remains essentially steady (at P[empty]≈−150 µW) while the ‘ovn_shn’ trace shows an increase (800<t<1500s) before stabilizing at P [*ovn_shn*] ≈−20 µW. The ‘Rot’ trace shows a smaller increase.

Because the offset of the instrument (100−200 μW typically, and different each time the instrument is turned on) is of the same order as the heating power we want to measure, we cannot use the absolute value of the heat flow in the steady state as our measurement. However, for experiments performed successively on the same day (as is the case for the traces of figure 5), we can assume an approximately constant offset, and use the difference between two traces in the steady state to extract a corresponding heating power. We call this measure ΔΔP. For the experiments of figure 5, the reference trace is ‘empty’, and averaging over the time interval 1500<t<2460s (the end of the recording) we find the extra heating power of the overexpressing cells compared to the silenced cells ΔΔP=140 μW, whereas ΔΔP[Rot]=25 μW. In fact, we can extend the averaging window to comprise the time during which the temperature controller is injecting heat to control the temperature, for example 500<t<2460s and obtain essentially the same values. The reason is that the cells’ heating power just adds to the temperature controller’s.

**Figure 6.**
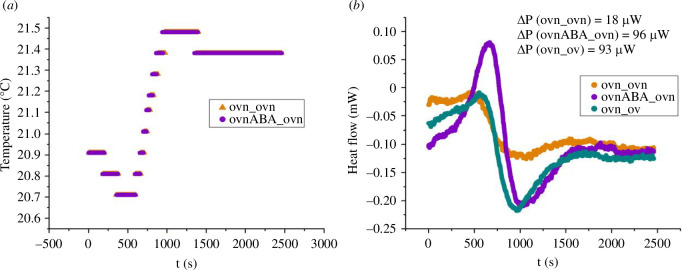
DSC measurements showing the effect of ABA on the thermogenesis of overexpressing cells. The ovn_ovn trace (ochra) serves as the reference, both crucibles containing the same mixture of overexpressing cells with nutrients. The ovnABA_ovn trace (purple) shows the effect of ABA activation, comparing overexpressing cells (with nutrients) with and without ABA. On comparison with the ovn_ov (green trace) (overexpressing cells with and without nutrients), we see that ABA activation roughly doubles the heating power generated by the cells. Quantitatively we find, for the ochra trace, ΔP[ovn_ovn]=18 μW while for the purple trace ΔP[ovnABA_ovn]=96 μW and for the green trace ΔP[ovn_ov]=93μ W. In this example ABA activation results in an increase in heating power of (96−18) μW =78 μW for the cells with nutrients, comparable to the heating power of (93−18) μ W =75 μ W obtained by adding nutrients to the cells.

An alternative way to extract the heating power is to take, for an individual trace, the difference between the steady state value at long times and the value at the minimum. We call this measure ΔP. The reason is that, with no extra heating (the ‘empty’ trace) and the isothermal protocol, the temperature controller cycle is such that ΔP≈0 (see ‘empty’ trace in figure 6*b*). For the traces of figure 5*b* we find: ΔP[ovn_shn]=184μW, ΔP[Rot]=19 μW and ΔP[empty]=18 μW so that subtracting this latter reference value from the others we obtain consistent values from the ΔΔP and ΔP measures. The latter has the advantage of being independent of the instrument’s offset, and so it can be used to compare experiments performed on different days.

Figure 6 shows another group of experiments, where we compare overexpressing cells with nutrients versus the same (ovn_ovn: ochra trace), overexpressing cells with nutrients and 100 nM ABA versus overexpressing cells with nutrients, but no ABA (ovnABA_ovn: purple trace), and overexpressing cells with nutrients vs overexpressing cells without nutrients (ovn_ov: green trace).

For the traces of figure 6 we find ΔP[ovnABA_ovn]=96 μW, ΔP[ovn_ov]=93 μW, while ΔP[ovn_ovn]=18 μW. Subtracting this latter reference value, this measurement indicates that overexpressing cells activated with ABA produce an extra heating power of 78 μW compared to non-activated cells, under the same conditions. Since overexpressing cells produce 93−18=75 μW of heating power according to this measurement, ABA activation approximately doubles the heating power produced by overexpressing cells with nutrients. Taken together, the measurements of figures 5,6 show that $N=2.5\times 10^{6}$ overexpressing cells produce an extra heating power of order of magnitude ∼100 μW compared to silenced cells, and that the ABA-activated cells produce an additional ∼100 μW of heating power, as an order of magnitude, compared to non-activated cells.

**Figure 7.**
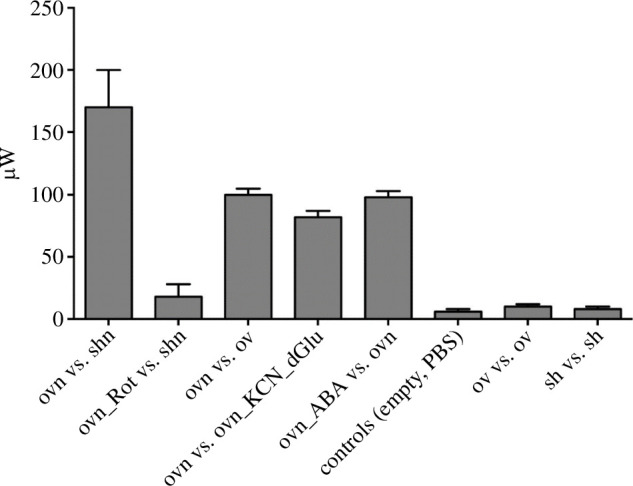
Summary of DSC measures of the power generated by LANCL1/2-overexpressing H9c2 cardiomyocytes. The measure reported is ΔP defined in the text, in microwatts. ov, LANCL1/2-overexpressing cells; sh, double-silenced cells; n, nutrients; dGlu, 2’-deoxy-glucose. Data are the mean ± s.d. of at least three measures, performed in independent experiments.

We performed a number of such experiments and controls. A summary of the results obtained from at least three different experiments for each condition is presented in figure 7.

Starved, LANCL1/2-overexpressing H9c2 (ov) or double-silenced cells (sh) were suspended in PBS+Ca^2+^/Mg^2+^ (2.5 ×10^6^ cells in 200 µl) in crucibles, which were tested pairwise in each experiment. Each experimental session included at least one control. Immediately before starting the temperature ramp of the DSC, and data recording, the nutrient mixture in PBS (*n*) or an equal volume of PBS alone, was added to the cells and the crucibles were capped and inserted in the DSC chamber. For oxphos inhibition, over-expressing cells were pre-incubated (or not) for 2 h with 0.03 mg ml^−1^ digitonin and 0.5 μM rotenone (Rot) + 0.5 μM antimycin A. To inhibit aerobic as well as anaerobic glucose metabolism, 3 mM KCN and 10 mM 2’-deoxy-glucose were added to over-expressing cells immediately before the start of analysis. Data shown are the mean ± s.d. from at least three experiments for each condition.

The heating power ΔP measured when overexpressing cells with nutrients were compared with overexpressing cells without nutrients (ΔP=93 μW) was quantitatively similar to the one observed when overexpressing cells with nutrients were compared with overexpressing cells without nutrients and also containing 2’-deoxy-glucose and KCN, to prevent oxidative metabolism (ΔP=89 μW). This observation indicates that starved cells contained no residual nutrients and only their addition at the start of the experiments provided cells with the substrates necessary for heat production. The main results are: (1) overexpressing cells produce a larger heating power compared to silenced cells (ΔP[ovn_shn]∼150 μW) and (2) ABA activated, overexpressing cells produce a larger heating power compared to non-activated (overexpressing) cells (ΔP[ovnABA_ovn]=95 μW). The last three bars in figure 7 represent controls. No significant increase of the heating power was observed in double silenced cells in the presence versus absence of ABA (not shown), in line with previous results indicating that double-silenced cells do not show the transcriptional and functional responses to ABA observed in overexpressing cells [5,17].

## 4. Discussion

Monitoring temperature fluctuations and/or heat production in living cells is of great interest to the biologist, as it informs on cell metabolism, mitochondrial respiration and uncoupling and may allow to identify molecular controllers of heat generation. Temperature monitoring on isolated cells has been obtained in the recent past mostly by indirect methods, via temperature-sensitive fluorescent or luminescent probes [18–22]. These methods suffer some limitations due to the short life-span of the fluorescent signal and its sensitivity to changing environmental cellular conditions. A recent and innovative study by Han *et al*. introduces an entirely different approach to temperature measurement (and not just ‘sensing’) by means of a sensor containing a Pd-Cr thin-film thermocouple, a freestanding Si3N4 platform and a dual-temperature control system: with this chip, the authors measured temperature fluctuations in human epithelial tumor cells (HeLa) (*of the order of magnitude of 0.3–1.5 K*) [23].

In our study, we measure the heating power produced by a population of cultured cells ($N=2.5\times 10^{6}$) by two different methods: directly by calorimetry, and indirectly by following the time course of the temperature fluctuations. Both methods are differential, the quantity measured being the difference (of heating power, or temperature, respectively) between a sample and a reference. We were able to compare samples of genetically modified cells, and draw conclusions on the corresponding physiology. Thus, we obtained a direct measurement of the heating power generated by the mitochondrial activity of rat H9c2 cardiomyocytes.

The measurements demonstrate a hitherto unknown role for the ABA/LANCL1−2 hormone-receptors system as a trigger of heat production in (cardio)myocytes. Cells overexpressing human LANCL1 and LANCL2 showed a significantly higher heat generation than cells double-silenced for the expression of endogenous LANCL1/2 and this difference was almost completely abolished by treatment with the electron transfer chain inhibitors rotenone and antimycin A (figure 7).

On the experimental side, a novel aspect of this study lies in the demonstration that a relatively simple apparatus based on low-cost, commercially available thermistor probes has sufficient sensitivity to allow measurement of heat production by a limited number of cells ($\sim 2\times 10^{6}$ cells), easily attainable not only with cultured cells, as in this study, but also with explanted tissues. Qualitative and quantitative confirmation of the experimental results obtained with the thermistor probes was obtained with measurements taken on the same cell types with a differential scanning calorimeter set on an isotherm mode of analysis. While the probes allowed to measure a temperature variation between two samples starting from a few seconds after nutrient addition to the starved cells, the DSC measurements had an approx. 15 min delay from the addition of nutrients, due to the time required for thermalization of the chamber containing the crucibles. This fact may explain the approximately threefold higher heat production measured with the temperature probes as compared with the DSC (approx. 400 versus approx. 150 µW, respectively) when comparing over-expressing versus double-silenced cells, both with nutrients. In the future, measurements with the temperature probes will allow to study the kinetics of how cell thermogenesis reacts to changing external conditions, and the dynamics of its regulation. Already the present measurements hint at an increased heating power in the first minutes after nutrients are added, compared to the long time steady state.

Both the temperature probes and the DSC measurements agree that heat generation by over-expressing cells is twice that of double-silenced cells. The calorimetry measurements show that ABA further increases heat production in over-expressing cells by approximately 100%.

How do these numbers compare with an estimate of the thermal energy produced by myocytes? We show in electronic supplementary material that based on ATP consumption, the average cell may produce 10 pW of heating power. This is a fraction of the 60 pW per cell we measure in the experiments with over-expressing cells. With these numbers, the heating power of the heart is negligible for overall thermal regulation in humans (see electronic supplementary material). The main source of heat in mammals is obviously skeletal muscle: indeed, the total muscle mass accounts for approximately 40% of body weight in males and 30% in females, i.e. approximately 100 times the percentage body weight of the heart. Shivering represents a reaction to cold and a means to increase muscle heat production through its involuntary contraction, sustained by an increased mitochondrial respiration and the ‘proton leak’ through the inner mitochondrial membrane, which is in part ‘basal’ but can be increased, by so-called ‘uncoupling’ proteins. Physical exertion increases body temperature through the same mechanism. A doubling of heat generation, as observed here in LANCL1/2-overexpressing versus -silenced myocytes, or in ABA-treated versus -untreated myocytes could result in a significantly increased muscle heat production *in vivo*. If muscle myocytes behaved similarly to LANCL1/2-overexpressing H9c2 (i.e. producing approx. 150 µW/$2.5\times 10^{6}$ cells = 60 pW cell^−1^ of heating power), total muscle myocytes (approx. $10^{13}$) would produce 600 W=2160 kJ h^−1^ of heating power. Brown adipose tissue (BAT) also produces heat, by means of the expression of tissue-specific ‘uncoupling’ proteins, which increase the amount of energy dissipated as heat in mitochondria. However, the contribution by BAT to human body heat production is limited by its reduced mass (compared with muscle) and by the fact that it is confined to discrete areas in the body, interestingly predominantly located in the mediastinum, around the heart and the major arterial vessels.

ABA-induced stimulation of the LANCL1 and LANCL2 hormone receptors as well as LANCL1/2 expression levels *per se* have been shown to stimulate mitochondrial biogenesis, O_2_ consumption and proton gradient dissipation in rodent skeletal myocytes [12], in human brown adipocytes [13] and in rat cardiomyocytes [17]. The approximately doubling of heat generation reported here for LANCL1/2-overexpressing versus double-silenced cardiomyocytes will likely be observed also on LANCL1/2-overexpressing versus double-silenced skeletal myocytes and brown adipocytes. In fact, transcription of several uncoupling proteins is significantly increased in LANCL1/2-overexpressing cells and further increases upon incubation of the cells with ABA: sarcolipin and UCP−3 in skeletal myocytes [12], the ADP/ATP translocator and UCP−1/3 in cardiomyocytes [17] and UCP−1 in brown adipocytes [13], indicating a possible systemic, whole-body increase of proton gradient dissipation controlled by tissue expression levels of the LANCL1/2 proteins.

Why would oxphos uncoupling be beneficial? By reducing reactive oxygen species (ROS) generation, an essentially unavoidable byproduct of respiration, mild uncoupling of mitochondrial oxidative phosphorylation shows potential to combat ROS-induced pathological conditions including obesity, neurodegenerative diseases, non-alcoholic fatty liver disease (NAFLD), diabetes and the metabolic syndrome and cardiovascular diseases [24]. Accumulating experimental evidence supports the conclusion that mild mitochondrial uncoupling, which naturally occurs in all tissues but can be increased pharmacologically or hormonally, is beneficial to the heart and prolongs rodents’ lifespan [25], which is arguably the sum of a combined beneficial effect on several organs and systems. Indeed, the mechanisms that alleviate proton and/or electron ‘jamming’ at the respiratory complexes, i.e. backwards electron transfer from reduced respiratory complexes to coenzymes/substrates [26,27] and proton leak through the inner mitochondrial membrane through ‘uncoupling’ proteins, allow the orderly flux of charges, maximize ATP production while at the same time minimizing ROS generation. It is noteworthy that overexpression of LANCL1/2 in murine L6 skeletal myoblasts [12], in rat H9c2 cardiomyocytes [17] and in human brown adipocytes [13] significantly increases transcription and expression of mitochondrial uncoupling proteins (ANT1, sarcolipin, UCP1 and UCP3), resulting in their logarithmic increase compared with double-silenced cells.

In conclusion, the present study provides to our knowledge the first demonstration that the ABA/LANCL system is involved in exothermic heat loss in mammalian cardiomyocytes and may open a new area of investigation into its role in thermogenesis. In this study, we used cardiomyocytes as a model cell system to investigate the possible role of the ABA/LANCL hormone/receptor system as a new regulator of cell heat production. Indeed, published observations on human iPS-derived brown adipocytes and on murine L6 myoblasts indicate that mitochondrial respiration, oxidative metabolism and OXPHOS uncoupling are higher in LANCL1/2-overexpressing compared with double-silenced cells [12,13], suggesting that the results shown here on cardiomyocytes could be extended to brown adipocytes and skeletal myocytes. Interestingly, overexpression of LANCL1/2 in human iPS-derived brown adipocytes significantly increases, while the double silencing conversely reduces, mRNA levels of β-adrenergic and of thyroid receptors [13], suggesting a possible interaction of the ABA/LANCL hormone/receptors system with hypothalamic-controlled thermal regulation and with thyroid hormones.

ABA administration to honeybee larvae has been shown to improve their survival at low temperatures, a clue that ABA, along with its conserved role as a stress hormone from plants to mammals, has other, as yet unexplored, functions in animal (and insect) physiology to surprise the open-minded researcher [28].

## 5. Conclusions

In conclusion, in this study we show that LANCL1/2-overexpressing rat cardiomyocytes produce twice the heat as double-silenced cells and that ABA further increases heat production by overexpressing cells.

Identification of the ABA/LANCL1−2 hormone/receptors system as a new trigger of (cardio)myocyte heat production may be of interest for several reasons:

— from a physiological point of view, it opens new areas of investigation into the interplay between this system and the thyroid- and beta-adrenergic-receptor mediated control of thermogenesis

— from a clinical point of view, it identifies the LANCL proteins as possible new targetable receptors to increase uncoupling of mitochondrial oxidative phosphorylation, an effect that the recent scientific literature agrees shows considerable potential to combat ROS-mediated pathological conditions including obesity, neurodegenerative diseases, non-alcoholicfatty liver disease, diabetes and cardiovascular diseases.

## Data Availability

All temperature recordings and DSC time traces used to generate the figures and measurements in the paper are contained in three annotated Origin Project files in the electronic supplementary material [29].
